# Management of a uterine serosal heterotopic pregnancy after in vitro fertilization in a woman with bilateral salpingectomy: A case report and literature review

**DOI:** 10.1097/MD.0000000000032551

**Published:** 2022-12-23

**Authors:** Ping-Ping Sun, Shu-Yi Dong, Jin-Long Xie, Kun-Kun Liu, Ai-Ping Guo

**Affiliations:** a The Reproductive Medicine Centre of Weifang People’s Hospital, Weifang, Shandong, China; b The Reproductive Endocrinology Department of Gaomi Maternity and Child Care Hospital, Weifang, Shandong, China.

**Keywords:** bilateral salpingectomy, ectopic pregnancy, heterotopic pregnancy, in vitro fertilization, uterine serosal pregnancy

## Abstract

Heterotopic pregnancy (HP) is defined as the simultaneous presence of intrauterine pregnancy and ectopic pregnancy (EP). HP after bilateral salpingectomy is extremely rare and may lead to serious complications if it is misdiagnosed and untreated timely. Here, we presented the first reported case of uterine serosal HP in a woman after assisted reproductive technology with bilateral salpingectomy because of bilateral tubal ectopic pregnancy.

**Patient concerns:**

A 27-years-old pregnant woman after in vitro fertilization with bilateral salpingectomy complained of a sudden onset of unprovoked abdominal pain, which was persistent and dull. She denied vaginal bleeding.

**Diagnoses:**

Serum beta-human chorionic gonadotropin levels are difficult to predict HP. Transvaginal ultrasonography demonstrated 1 gestational sac in the uterine cavity and 1 thick-walled cystic mass over the upper of the uterus, with a large amount of fluid in the Pouch of Douglas. Emergency laparotomy revealed a uterine serosal pregnancy combined with intrauterine pregnancy.

**Interventions:**

This patient was successfully managed via emergency laparotomy to remove residual tissue and repair the rupture of the uterine serosal pregnancy.

**Outcomes:**

At postoperative 4 days, repeat transvaginal ultrosonography presented 1 intrauterine gestational sac with a visible fetal bud and cardiac tube pulsation. Now the patient recover well and is in an ongoing pregnancy.

**Lessons:**

It is noteworthy that HP/ectopic pregnancy is still not prevented after bilateral salpingectomy. In cases of multiple embryo transfer, even if intrauterine pregnancy has been established, it is important to rule out HP/ectopic pregnancy in time. Early diagnosis and early management can significantly improve clinical outcomes.

## 1. Introduction

Heterotopic pregnancy (HP) is a pathological pregnancy that is characterized by the simultaneous presence of intrauterine pregnancy and ectopic pregnancy (EP).^[1]^ It is estimated that most EP occurs in the ampulla of fallopian tubes, and less in the other sites, such as an interstitial pregnancy, cervical pregnancy, intramural pregnancy, and ovarian pregnancy. The incidence of HP in the general population who conceive naturally is extremely low,^[1,2]^ and now the incidence is on a marked increase with the widespread use of assisted reproductive technology (ART) for infertile couples worldwide.^[2,3]^ ART increases not only the risk of HP but also the complexity of the type of EP. Some EP that is rare or even generally considered impossible to occur in natural pregnancy appears after ART. Here we present a rare case of HP, that is concomitant intrauterine and uterine serosal pregnancy following in vitro fertilization in a woman with bilateral salpingectomy. To our knowledge, such a case has not been reported before.

## 2. Case report

**Figure 1.**
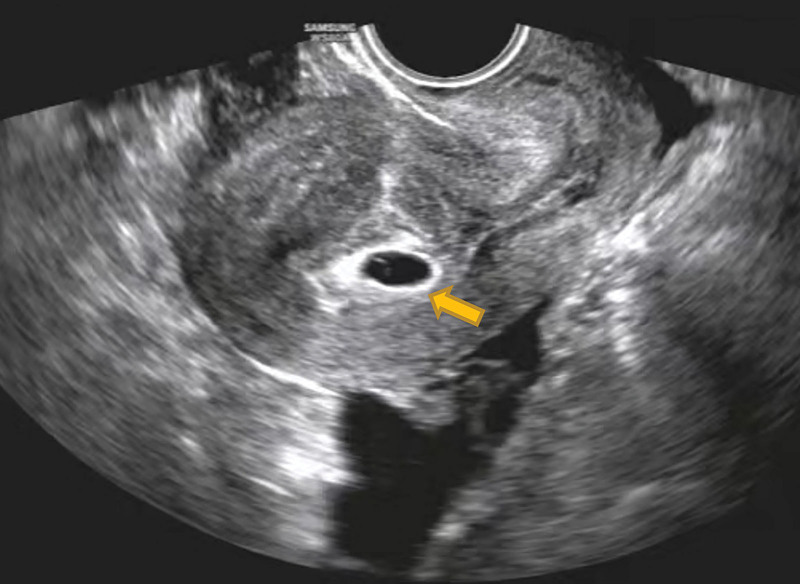
One intrauterine gestational sac with an amount of fluid measured about 8.8 × 4.7 cm in the Pouch of Douglas.

**Figure 2.**
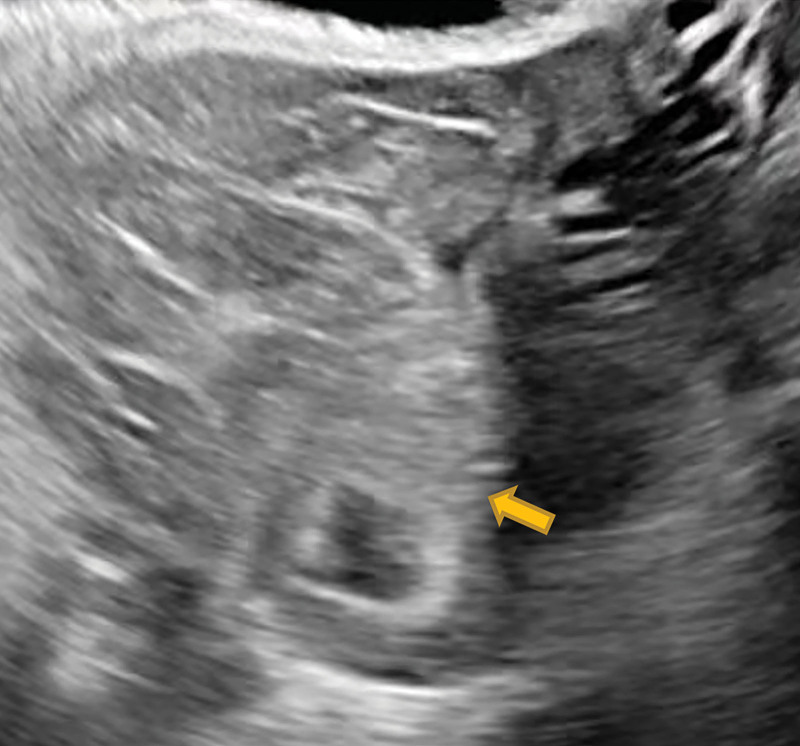
There was a thick-walled cystic mass measuring about 2.3 × 2.1 cm, over the upper left of the uterus.

**Figure 3.**
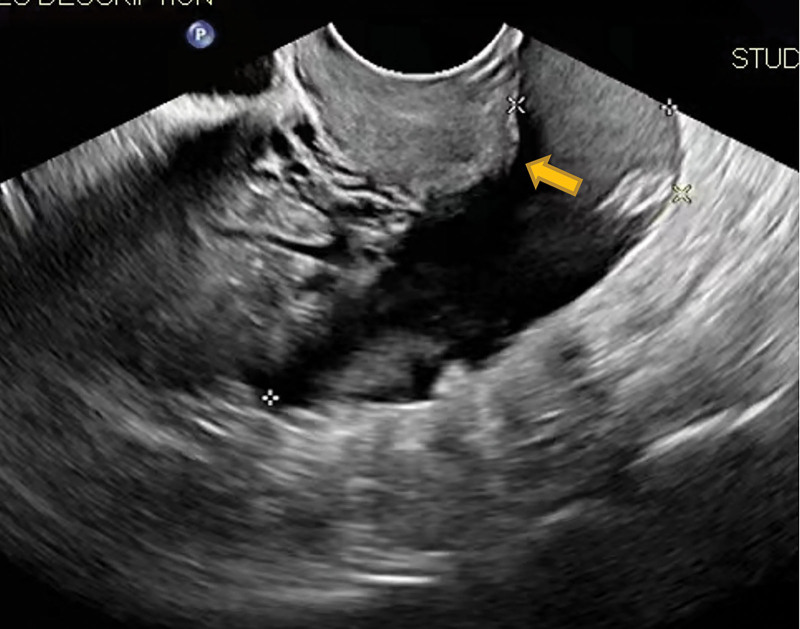
There was a large amount of free fluid within the Pouch of Douglas, approximately measuring 9.0 × 4.7 cm in extent.

**Figure 4.**
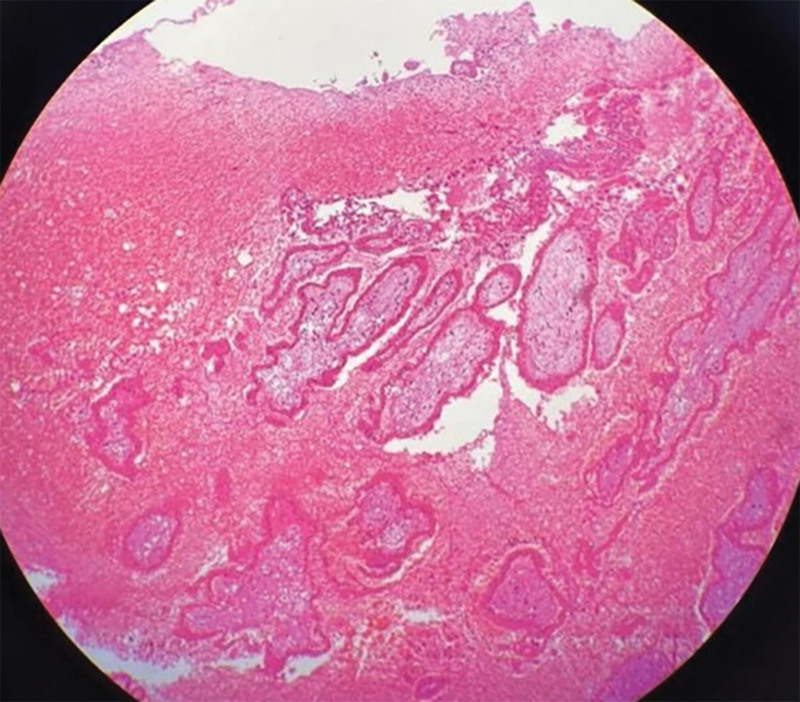
The pathological examination showed villi tissue in the specimen.

The patient was a 27-years-old woman, gravida 2, para 0, ectopic pregnancies 2, known to have bilateral salpingectomy for ectopic pregnancies. She was referred to our reproductive center with a request for in vitro fertilization. The patient received 14 oocytes and canceled the fresh embryo transfer due to abdominal distension. At last 2 cleavage-stage embryos and 1 blastocyst were frozen. Then the patient transferred 2 cleavage-stage thawed embryos after 2 months. The transplant was performed without the use of the catheter core under abdominal ultrasound guidance, but only 1 embryo was transferred into the uterine cavity. So the patient was reintubated for a second ET, which went very well this time. The patient’s serum beta-human chorionic gonadotropin (β-hCG) level was 1980 mIU/mL 14 days after ET. However 19 days after ET, the patient complained of a sudden onset of unprovoked abdominal pain, which was persistent and dull. On physical vaginal examination, the cervix showed positive painful lifting, and the uterus was enlarged slightly and had pressure pain. The bilateral adnexal region also had positive pressure pain but no palpable masses. Transvaginal ultrasonography (TVUS) demonstrated 1 intrauterine gestational sac and an amount of fluid measured about 8.8 × 4.7 cm in the Pouch of Douglas, with no abnormalities in the bilateral adnexal region (Fig. 1). Routine blood chemistry showed WBC 14.8 × 10^9^/L; neutrophil ratio 88.4%; RBC 4.18 × 10^12^/L; HB 136 g/L; erythrocyte pressure 42%. The patient was treated with antibiotics and had diffuse lower abdominal pain for 2 days duration. Again transvaginal ultrosonography demonstrated a thick-walled cystic mass measuring about 2.3 × 2.1 cm over the upper left of the uterus in addition to 1 intrauterine pregnancy (Fig. 2). And ultrasonographic evidence of pelvic hemoperitoneum was present, with a large amount of free fluid within the Pouch of Douglas, approximately measuring 9.0 × 4.7 cm in extent (Fig. 3). The patient underwent emergency laparotomy due to the high suspicion of EP. Entering the abdominal cavity, a hemoperitoneum of approximately 400 mL was noted and a 1-cm “crater-like” rupture was found on the left serosa of the uterine fundus. The bilateral uterine horns were normal. Bilateral tubal absences and bilateral ovaries were grossly normal. Grossly, the rupture was superficial, which was covered by a blood clot and was confined within the serosa, not reaching the myometrium and the uterine cavity. So the patient was tentatively diagnosed with a uterine serosal pregnancy combined with intrauterine pregnancy. After removing the abnormal tissue around the rupture, the uterine scar was meticulously sutured using absorbable sutures and the specimen was sent for pathology. The operation went well and the estimated blood loss was minimal. The postoperative recovery was uneventful and the patient was continued to give progesterone treatment. At postoperative 4 days, repeat transvaginal ultrosonography presented 1 intrauterine gestational sac with a visible fetal bud and cardiac tube pulsation. Histopathology of the tissues confirmed the uterine serosal pregnancy, concordant with the intra-operative diagnosis (Fig. 4). At the time of writing the patient is in an ongoing pregnancy.

## 3. Discussion

### 3.1. Incidence

HP is extremely uncommon and its incidence varies markedly in different populations. The incidence of HP in all spontaneous pregnancies is less than 1 in 30,000,^[2]^ while the incidence of HP is approximately increasing to a rate of 0.1% to 1% following ART.^[1,2,4]^ Meanwhile, ART also increases the chance of rare implantation sites of EP, such as abdominal pregnancy,^[5]^ interstitial pregnancy,^[6]^ ovarian pregnancy,^[7,8]^ and even intramural pregnancy(IMP).^[9]^ IMP is extremely rare and was first described by Lu et al,^[10]^ accounting for fewer than 1% of total ectopic pregnancies.^[11]^ Uterine serosal pregnancy is considered a variant of IMP^[12]^ and differs from the classic definition of IMP. In uterine serosal pregnancy, the gestational sac was surrounded by the serosa of the uterus, lacking distinct myometrial tissue and no linking with the fallopian tubes, the endometrial cavity, or the round ligament.^[13]^ Uterine serosal pregnancy is rare, with only a few cases reported to date.^[12–14]^ Here it’s reported an extremely unusual case of uterine serosal heterotopic pregnancy following ART in a woman with bilateral salpingectomy, which is the first known case reported to date.

### 3.2. Etiology

Theoretically, HP/EP can be avoided after bilateral salpingectomy, but clinical data show that there is still a risk of HP/EP the following ART after bilateral salpingectomy.^[5,15,16]^ The embryo may wander for 3 to 4 days before implantation after ET, during this time it may implant extrauterine. The exact mechanism of the extrauterine implantation after bilateral salpingectomy is still a mystery. At present, there are several possible explanations.

One possible mechanism for the extrauterine implantation of the embryo is the difficulty of ET and even perforation of the uterus during ET.^[8]^ However, this explanation does not fit this case. ET was applied using a very soft and flexible catheter under direct vision with intensive transabdominal ultrasound guidance, so any perforation would have been seen in real-time, even though the patient had a little trouble during ET. Furthermore, the presence of a concomitant intrauterine pregnancy in this patient suggested a favorable uterine environment, which excludes the possibility of uterine damage.

Another possible proposed mechanism is the migration of the embryo to extrauterine via lymphatic vessels, similar to the metastasis of endometrial cancer.^[5]^ The embryo was deposited deep in the endometrium and subsequently entered the lymphatic vessels, spreading outside the uterine along the uterine lymphatic channels. Therefore it was seen for the embryo implant around the retroperitoneal artery and formed retroperitoneal EP.^[17,18]^ In this case, the extrauterine embryo was implanted in the serosal of the uterine fundus, which was not the lymphatic channel of the uterus, so the mechanism of lymphatic pathways was impossible to explain uterine serosal implantation of the embryo.

The third possibility and more probable mechanism is a microscopic fistulous tract at the stump of the fallopian tube, which is connected to the abdominal cavity, even though a tract was not seen with the naked eye at the time of surgery. Theoretically compared with women whose salpingectomy was performed routinely for permanent contraception, women who had salpingectomies at the time of potentially distorted anatomy (for EP, suspected PID, or hydrosalpinx) had a higher risk of incomplete tubal resection and subsequent subperitoneal fistula formation.^[19]^ The embryo can migrate to the abdominal cavity via a tubal microscopic fistula and be implanted in nearby tissues. In this case, the sensitivity of the constriction of myometrium and intensity of the endometrial waves may have been affected by the fact that 1 embryo was left out at the time of transfer and was retransferred. Intrauterine embryos maybe change their travel pattern and enter the abdominal cavity via the microscopic fistula of the fallopian tube stump. Coincidentally, the tubal stump was right near the fundus of the uterus, so this migrating embryo was implanted in the serosal of the uterine fundus and formed the uterine serosal pregnancy. And the re-transferred embryo maybe implates in the uterus, creating an intrauterine pregnancy.

### 3.3. Diagnosis

Concomitant intrauterine and uterine serosal pregnancy is a very rare type of HP that lacks typical clinical symptoms and is easily missed and misdiagnosed. Now the diagnosis of HP mainly relies on β-hCG levels and transvaginal ultrasound. It has been suggested that low beta-human chorionic gonadotropin (β-HCG) levels 14 days after ET are associated with the possibility of EP.^[1]^ Actually, β-HCG levels are difficult to predict HP because intrauterine pregnancy can lead to β-HCG levels appropriate increase. In this case, the patient’s serial β-HCG levels were not low. TVUS is thought to be an important aid in the diagnosis of HP, which can assess the whole pelvis and discover ectopic lesions. The sensitivity of TVUS for the diagnosis of EP can vary from 73% to 93% and mainly depend on the expertise of the ultrasonographer,^[4]^ the gestational age, and the type of EP.^[20]^ If the pelvis cannot be fully scanned or the location of the embryo implantation is rare, TVUS also has a low sensitivity and misses the diagnosis. In this case, the first TVUS of the patient demonstrated there was 1 intrauterine gestational sac and no extrauterine abnormal mass, even though a large volume of fluid in the Pouch of Douglas. Unfortunately, it failed to give any attention and lead to a missed diagnosis. Although a scant amount of pelvic fluid can be normal, the significant pelvic fluid should raise suspicion for hemoperitoneum following EP.^[20]^ Also, the abdominal pain in this patient was precisely related to a large amount of fluid in the Pouch of Douglas. It was not until again TVUS that detected a cystic mass in the upper left of the uterus and a large amount of pelvic fluid, HP was highly suspected. Then emergency laparotomy was performed and HP was confirmed. It can be seen that early diagnosis of HP in rare sites, especially after bilateral salpingectomy, remains still elusive and challenging.

### 3.4. Management

The management of HP largely depends on the state of coexisting intrauterine pregnancy. If the intrauterine pregnancy is viable, the optimal treatment of HP is the minimally invasive approach to preserve the intrauterine pregnancy. Removal of the ectopic lesion via laparoscopy or laparotomy is the most preferred option^[1]^, in this case. Conversely, if intrauterine pregnancy is not viable, then there are various options for the treatment of HP, ranging from conservative medication to surgical treatment depending on the patient’s status.^[21]^ Moreover, like this patient, because some EP of rare locations are difficult to detect by ultrasound, therefore diagnostic surgery should be considered as a first-line intervention to both clarify the diagnosis and give treatment. Another, patients with high suspicion of EP should be immediately transferred for surgery if she has peritoneal signs or hemodynamic instability.^[22]^ Due to the surgical intervention timely in this patient, she not only had a definitive diagnosis of HP (concomitant uterine serosal and intrauterine pregnancy) but also had a better prognosis.

### 3.5. Prevention

After bilateral salpingectomy, the patient will choose ART for assisted reproduction. It is noteworthy that HP/EP is still not prevented after bilateral salpingectomy, therefore clinicians still need to be vigilant. Primary preventive measures for HP/EP are limited, thus it is important to recognize the risk factors for HP/EP. Some studies have shown that risk factors for HP/EP after ART were found to be mainly related to previous treatment of EP, the type of embryos transferred, the number of embryos transferred, and the transfer technique.^[23,24]^ When performing the bilateral salpingectomy, surgeons must enhance electrodestruction or suture of the fallopian tube stump, which can close the residual interstitial canal lumen of the uterus and avoid transfer of the embryo to extra-uterine implantation via the fistula of the fallopian tube stump.^[25]^ Studies in ART have found that frozen embryos transfer can reduce the incidence of EP more than fresh embryo transfer,^[26]^ that blastocyst transfer also can reduce the incidence of EP compared to cleavage embryo transfer,^[27]^ that the incidence of EP was significantly lower when the thickness of the endometrium is 8 to 12 mm for transplantation,^[28]^ and that single embryo transfer also can minimize the risk of EP.^[29]^ Another study confirmed that the development of EP following ART has been related to the embryo transfer technique. Pressure and speed of the embryo injection into the uterine cavity, the volume of the transfer medium, and the depth of the transfer catheter during ET may affect the movement of the embryo and the placement of implantation.^[24]^ So such as those with EP history, it may be a reasonable clinical practice to choose single frozen blastocyst transfer at the appropriate endometrial thickness. Also, it needs to take into account the details of the transfer technique, such as the placement of the embryo at least 10 mm close to the uterine fundus^[30]^ and reduction of injected pressure and volume of the medium are effective primary preventive measures to decrease the incidence of HP/EP in ART. In practice, however, secondary and tertiary prevention of HP/EP is more achievable in clinical practice. A series of determinations of serum β-hCG, even as early as 11 to 12 days after ET, and early TVUS is predictive of EP.^[24]^ Be alert to the possibility of EP when vaginal spotting or abdominal pain. Also, surgical intervention should be decisively undertaken when the patient’s hemodynamic instability. In short, early diagnosis and early intervention not only have a good prognosis but also prevent the occurrence of serious complications.

## 4. Conclusion

The incidence of HP is relatively lower, uterine serosal pregnancies are extremely rare, and the case of intrauterine pregnancy combined with uterine serosal pregnancy following bilateral salpingectomy has not been reported. It is because of this rarity that misdiagnosis can occur in clinical work, so the clinician needs to observe the smallest detail and focus on differential diagnosis. In this case, with a history of bilateral salpingectomy and a visible intrauterine pregnancy, it is extremely easy to omit HP, and therefore the likelihood of clinical omission is high.

With the widespread use of ART, rare HP may become more common. Always be aware of the possibility of EP in pregnant women with abdominal pain, even in patients with bilateral salpingectomy. In cases of multiple embryo transfer, even if intrauterine pregnancy has been established, it is important to rule out EP in time. Early diagnosis and early management can significantly improve clinical outcomes.

## Author contributions

**Conceptualization:** Ai-Ping Guo, Ping-Ping Sun.

**Data curation:** Ping-Ping Sun.

**Formal analysis:** Ping-Ping Sun.

**Investigation:** Ping-Ping Sun, Shu-Yi Dong, Jin-Long Xie.

**Software:** Ping-Ping Sun.

**Supervision:** Ai-Ping Guo.

**Validation:** Ping-Ping Sun, Shu-Yi Dong, Jin-Long Xie, Kun-Kun Liu, Ai-Ping Guo.

**Writing – original draft:** Ping-Ping Sun.

**Writing – review & editing:** Ping-Ping Sun, Ai-Ping Guo, Shu-Yi Dong, Jin-Long Xie, Kun-Kun Liu.
